# The long-term clinical outcomes of intravascular ultrasound-guided versus angiography-guided coronary drug eluting stent implantation in long *de novo* coronary lesions: A systematic review and meta-analysis

**DOI:** 10.3389/fcvm.2022.944143

**Published:** 2022-08-04

**Authors:** Shen Wang, Changzai Liang, Yue Wang, Shuaifeng Sun, Yue Wang, Min Suo, Maomao Ye, Xinjian Li, Xinyan Liu, Meng Zhang, Xiaofan Wu

**Affiliations:** ^1^Department of Cardiology, Beijing Anzhen Hospital, Capital Medical University, Beijing, China; ^2^Department of Cardiology, Aerospace Center Hospital, Beijing, China

**Keywords:** intravascular ultrasound, angiography, drug-eluting stent, outcomes, long *de novo* coronary lesions

## Abstract

**Background:**

No meta-analysis has been conducted to compare the long-term clinical outcomes of intravascular ultrasound (IVUS)-guided versus angiographic-guided drug-eluting stent implantation in patients with long *de novo* coronary lesions. We attempted to compare the efficacy and safety of IVUS guidance versus angiography guidance in percutaneous coronary intervention (PCI) for long *de novo* coronary lesions.

**Materials and Methods:**

We performed a detailed meta-analysis from four randomized controlled trials (RCTs) and one observational study to compare long outcomes of IVUS versus angiography in guiding coronary stent implantation with long *de novo* coronary lesions defined as coronary stenosis which need stent implantation >28 mm in length. Data were aggregated for the endpoints measure using the fixed-effects model as pooled odds ratio (OR) with 95% confidence intervals. Clinical outcomes included major adverse cardiovascular events (MACE), all revascularization, including target lesion revascularization (TLR) and target vessel revascularization (TVR), all myocardial infarction (MI), all-cause death, and stent thrombosis (ST). Cochrane Library, Embase, PubMed, and Web of Science were searched.

**Results:**

Four RCTs and one observational study were included in our study with 3,349 patients (IVUS guidance = 1,708; Angiography guidance = 1,641). With mean follow-up of 2 years, the incidence of MACE, all myocardial infarction, all revascularization and stent thrombosis were significantly lower in IVUS-guided DES implantation of patients with long *de novo* coronary lesions than in angiography-guided patients; MACE [OR 0.41; 95% confidence interval (CI), 0.29–0.58; *p* < 0.00001], all myocardial infarction (OR 0.23; 95% CI, 0.09–0.58; *p* = 0.002), all revascularization (OR 0.48; 95% CI, 0.36–0.66; *p* < 0.00001), stent thrombosis (OR 0.32; 95% CI, 0.11–0.89; *p* = 0.03). There was no significant difference in all-cause mortality between the two groups (OR 0.82; 95% CI, 0.55–1.23; *p* = 0.34).

**Conclusion:**

During mean follow-up of 2 years, the incidence of MACE, stent thrombosis, all myocardial infarction and revascularization in patients with long *de novo* coronary lesions under IVUS-guided PCI were significantly lower than angiography-guided PCI, and there were no statistically significant differences in all-cause mortality.

## Introduction

Intravascular ultrasound (IVUS) provides anatomic information regarding the coronary artery lumen, wall, and plaques, which can help the accurate evaluation of lesion characteristics with vessel sizing. In addition, IVUS can not only detect underexpansion, improper adhesion, stent rupture or edge stripping after stent implantation, but also detect percutaneous coronary intervention (PCI)-related complications (including in-stent restenosis and in-stent thrombosis). Thus, through further intervention based on these IVUS findings, stent optimization can be achieved, causing the improved clinical outcomes. Current guidelines recommend the use of IVUS to optimize stent implantation for select patients (Class of recommendation IIa, Level of evidence B) (1, 2). In the era of drug-eluting stents (DES), stent segment length was an independent predictor of restenosis and stent thrombosis. It is not clear whether the IVUS-guided PCI strategy can lead to better clinical outcomes in patients undergoing DES implantation for long *de novo* coronary lesions. Lastly, meta-analyses compared the IVUS-guidance and angiography-guidance during PCI, including complex lesions, such as left main lesions, chronic total occlusions, and bifurcation lesions (3–6). But there have been no meta-analysis to compare long-term clinical outcomes of IVUS versus angiography-guided PCI within in long *de novo* coronary lesions. Thus we performed this meta-analysis.

## Materials and methods

### Literature search

**FIGURE 1:**
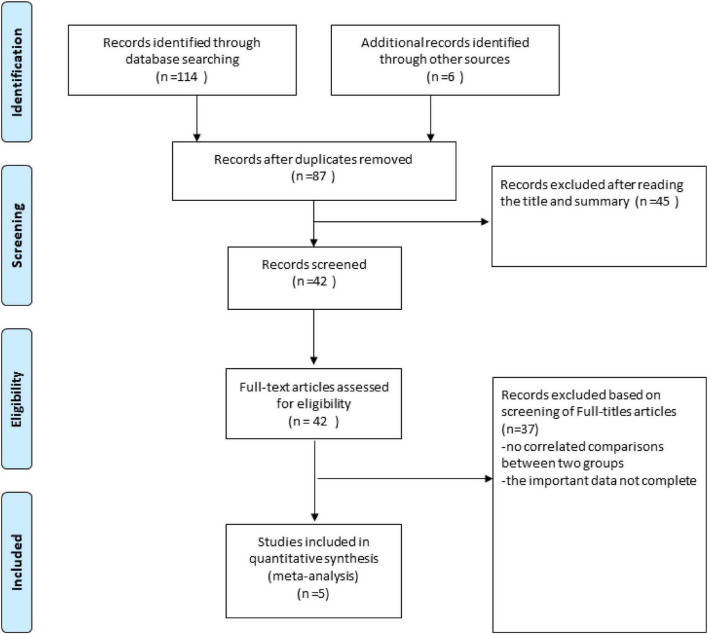
Flow diagram of the search strategy for systematic review and meta-analysis.

The PubMed, Cochrane Library, Medline and EMBASE from there date of inception to March 15, 2022. The combinations of the several relevant terms were used to promise all studies were included in the literature search process: “ultrasonography, intravascular,” “intravascular ultrasound,” “intravascular ultrasound-guided,” “IVUS,” “IVUS-guided,” “CAG,” “Angiography,” “Angiography -guided,” “CAG-guided,” “drug-eluting stent,” “sirolimus-eluting stent,” “paclitaxel-eluting stent,” “everolimus-eluting stent,” “zotarolimus-eluting stent,” “stent,” “DES,” “PCI,” “percutaneous coronary intervention” and “long *de novo* coronary lesions,” “large *de novo* coronary lesions,” “diffuse long lesions.” The title and abstract, as well as the full text of the original report in the study, were independently screened and verified by two researchers (CZL and SW) (Figure 1).

### Inclusion and exclusion criteria

The inclusion criteria were as follows: (1) Adults must be 18 years of age or older to undergo PCI with DES; (2) randomized controlled trials (RCTs) and observational studies must include IVUS-guided and CAG-guided DES implantation comparisons with clinical follow-up of at least 6 months. (3): Coronary artery lesions only include long *de novo* lesions (stent length ≥ 28 mm). The exclusion criteria were as follows: (1): The patients presenting with ST-segment elevation myocardial infarction (MI) and who had coronary lesions involving chronic total occlusion (CTO), localized left main lesions, in-stent restenosis lesion and bifurcation lesions require two stents. (2) Patients with severe left ventricular dysfunction (ejection fraction < 30%), cardiogenic shock, and neoplastic disease.

### Data extractions and quality assessments

Two reviewers (CZL and SW) reviewed all relevant articles for assessing their eligibility. The following data were extracted from each included study: the first author’s name of the trial, publication year, baseline demographics, procedural characteristics, and clinical outcomes during follow-up. The third reviewer (YW) resolved disagreements. Using the Cochrane Collaboration Risk of Bias tool to assess the quality of all RCTs.

### Study endpoints

The primary endpoint of this study was the incidence of major adverse cardiac events (MACE), including cardiac death, target lesion-related myocardial infarction, or ischemia driven target-lesion revascularization. Secondary outcomes included all myocardial infarction, all-cause mortality and stent thrombosis and all revascularization [including target-lesion revascularization (TLR) and target-vessel revascularization (TVR)]. MACE was defined as a composite of cardiac death, MI, TLR according to the definition of the Academic Research Consortium (7).

### Statistical analysis

For baseline data, continuous variables were measured using the unpaired *T*-test of two samples of students, using mean ± SD, and classification variables were measured using the ratio of Chi-square statistics. All endpoints were assessed by odds ratios (ORs) with 95% confidence intervals (CIs). Using chi-square tests and *I*^2^ statistics to assess the statistical heterogeneity between RCTs. When the *p* value of *Q* test was < 0.10 and/or the *I*^2^ was ≥ 50%, significant heterogeneity was considered and a random-effects model would be selected. If not, the fixed-effects model was used instead. All reported *p*-values were two-tailed, and *P*-values < 0.05 were considered statistically significant. Egger test and funnel plot were used to assess potential bias. Statistical analysis was performed using Review Manager 5.3 software.

## Results

### Studies included

After screening through 87 articles, finally, a total of four RCTs and one observational study with 3,349 participants were included (1,708 patients in the IVUS guidance group and 1,641 patients in the angiography guidance group) (5, 8–10).

### Patient characteristics and procedural characteristics

Table 1 shows the baseline characteristics of the four RCTs and one observational study included in our study. All studies were followed for a minimum of 1 year and a maximum of 3 years. There were no statistically significant differences in baseline characteristics between the IVUS-guided and angiography-guided groups. Table 2 shows the procedural characteristics between the IVUS-guided and angiography-guided groups. The procedural characteristics, including access site, lesion characteristics, and stent length and target lesion vessel are summarized in Table 2. There was no statistical difference between the two groups. The most common vascular puncture route in both groups was the radial artery route.

**TABLE 1:** Study design, baseline characteristics of the included studies.

References	Design	Sample size (*N*) (IVUS/CAG)	F/U (years)	Age (years)	Male (%)	Smoker	DM	Hypertension	Hyperlipidemia	LVEF (%)	Prior PCI	Prior CABG	Prior MI
Oemrawsingh et al. (8)	RCT	144 (73/71)	1 year	61/63	71 (96.0)/72(94.7)	40/53	16/21	27/30	61/62	NA	NA	NA	NA
Ahn et al. (9)	Observational	85 (49/36)	2 years	65/65	22 (61.1)/30 (61.2)	14/16	11/13	20/25	9/14	56/54	3/1	0/0	2/2
Kim et al. (10)	RCT	543 (269/274)	1 years	62.8/64.5	197 (66.3)/130 (52.8)	67/38	90/77	187/156	190/144	55.2/53.9	11/8	3/11	35/29
Hong et al. (5)	RCT	2577 (1289/1288)	3 years	65/65	912 (71)/917 (71)	364/366	442/451	881/867	787/784	62/61	181/182	28/22	92/102

MI, myocardial infarction; RCT, randomized control trial; DM, diabetes mellitus; F/U, follow-up; LVEF, left ventricular ejection fraction; PCI, percutaneous coronary intervention. All the data is arranged in IVUS/CAG format.

**TABLE 2:** Procedural characteristics.

Variable	Oemrawsingh et al. (8) IVUS/CAG	Ahn et al. (9) IVUS/CAG	Kim et al. (10) IVUS/CAG	Hong et al. (5) IVUS/CAG
Coronary arteries				
RCA, *n*	51/41	14/18	65/50	367/425
LAD, *n*	39/38	29/16	191/161	825/805
LCX, *n*	10/21	6/2	41/35	244/259
#Lesion length, mm	29/27	68/60	29.8/30.5	34.8/34.6
#Stent length, mm	42/35	74/66	33/31	60.81/60.86
#Stent number, n	1.4/1.1	2.8/2.2	NA	2.11/2.15
#Stent, diameter, mm	NA	3.00/2.87	NA	NA
#Reference vessel diameter, mm				
Pre-intervention	2.95/2.96	2.8/2.9	2.82/2.79	2.81/2.81
Post-intervention	3.45/3.24	2.9/3.0	NA	2.96/2.89
Follow-up	2.84/2.74	3.0/3.0	NA	NA
#Diameter stenosis, %				
Pre-intervention	65/65	75/83	NA	NA
Post-intervention	12/13	7/13	NA	NA
Follow-up	38/45	20/56	NA	NA
Restenosis, %	23/46	NA	NA	NA
#MLD, mm				
Pre-intervention	1.02/0.99	0.7/0.5	0.97/0.90	0.83/0.84
Post-intervention	3.01/2.80	2.8/2.6	2.58/2.51	2.56/2.49
Follow-up	1.82/1.51	2.4/1.3	NA	NA
Acute gain	2.04/1.81	2.1/2.1	1.55/1.56	NA
Types of DES	AVE GFX-XL (Medtronic/AVE)	Sirolimus/Paclitaxel Everolimus/Zotarolimus-eluting stent	Everolimus/Zotarolimus-eluting stent	Everolimus-eluting stent

DES, drug-eluting stent; LAD, left anterior descending artery; LCX, left circumflex artery; mm, millimeters; RCA, right coronary artery; NA, not available; MLD, Minimum lumen diameter; #, these data are mean value.

### Clinical outcomes

**FIGURE 2:**
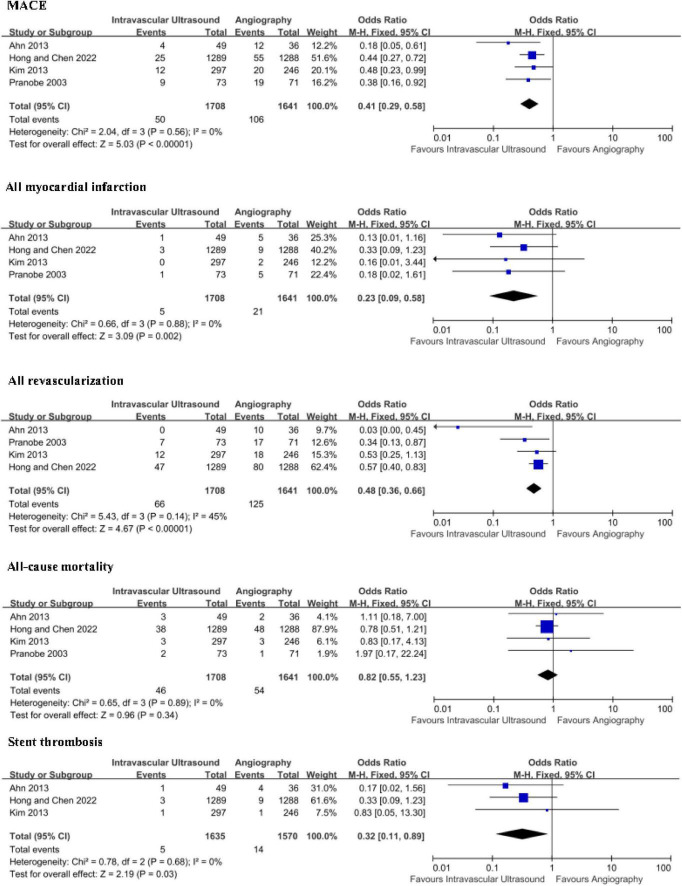
All long clinical outcomes forest plot–random effect including major adverse cardiovascular events (MACE); All myocardial infarction; All revascularization; All-cause mortality; Stent thrombosis.

Figure 2 shows the clinical outcomes of the two groups between IVUS-guided PCI and angiography-guided PCI. The definition of MACE was slightly consistent across all studies, including cardiac death, myocardial infarction, and TLR (7).

In terms of MACE, IVUS-guided PCI had statistically significant lower incidence than angiography-guided PCI [OR 0.41; 95% confidence interval (CI), 0.29–0.58; *p* < 0.00001] with no statistical heterogeneity among the included studies (*I*^2^ = 0%; *P* = 0.56) (Figure 2).

In the analysis of all myocardial infarction, the risk of all myocardial infarction was statistically significant lower in IVUS-guided PCI group (OR 0.23; 95% CI, 0.09–0.58; *p* = 0.002). There was no statistical heterogeneity among all studies (*I*^2^ = 0%; *P* = 0.88) (Figure 2).

Four studies were applied to the analysis of all revascularization. The result of all revascularization was statistically significant lower in IVUS-guided PCI group (OR 0.48; 95% CI, 0.36–0.66; *p* < 0.00001) with no heterogeneity (*I*^2^ = 45%; *P* = 0.14) (Figure 2).

As for all-cause mortality, all studies demonstrated that IVUS-guided PCI was not superior to angiography-guided PCI, and there was no significant difference in all-cause mortality between the two groups (OR 0.82; 95% CI, 0.55–1.23; *p* = 0.34). There was no statistical heterogeneity among the four RCTs (*I*^2^ = 0%; *P* = 0.89) (Figure 2).

Data on stent thrombosis (definite, probable, and possible) was reported in four studies. With a mean follow-up of 2 years, the incidence of ST in the two groups was 0.31% (five cases of IVUS-guided Group) and 0.89% (14 cases of angiography-guided group). There was significant reduction in the risk of ST in the IVUS-guided PCI group compared with the angiography-guided PCI group (OR 0.32; 95% CI, 0.11–0.89; *p* = 0.03), and there was no statistical heterogeneity (*I*^2^ = 0%; *P* = 0.68) (Figure 2).

## Discussion

This meta-analysis of 3,349 patients from four RCTs and one observational study showed that the IVUS-guided PCI statistically significant reduce in long-term clinical outcomes (MACE, all myocardial infarction, all vessel revascularization, and stent thrombosis) comparing with angiography-guided DES during a median follow-up of 2 years. But there are no statistically significant differences in all-cause mortality between IVUS-guided and angiography-guided PCI.

To our knowledge, this is the first meta-analysis from four RCTs and one observational to compare long-term clinical outcomes of IVUS versus CAG in directing PCI of long *de novo* coronary lesions. Previously less meta-analyses have explored the impact of IVUS versus CAG in guiding PCI but mixed with complex lesions (11–13). Recently, with the release of the 3-year clinical outcomes of IVUS-XPL and ULTIMATE trials, whose conclusion was that using IVUS guidance in DES with long *de novo* lesions improved long-term patient cardiac survival than CAG guidance (5). Our results also confirm the similar long clinical outcomes of IVUS versus CAG guidance in DES of long *de novo* lesions.

Longer lesion length is a well-known independent risk factor for stent failure including restenosis and stent thrombosis, which may be associated with under stent expansion (7, 11, 12). Therefore, the achievement of sufficient lumen area by IVUS may be imperative. More interestingly, however, whether or not IVUS-optimized stent implantation is performed further determines the long-term clinical benefit. On one hand, as for quantitative measurements of native lesion, IVUS can accurately represent the sizes (dimensions) or the composition of coronary plaque and luminal narrowings than angiography. On the other hand, as for qualitative assessment of native lesion, IVUS can grossly separate lesions into subtypes according to echo density and the presence or absence of shadowing and reverberations. Such as calcium, dense fibrous tissue, lipid, smooth muscle cells, thrombus, etc. Depending on the nature of the plaque, the operators may decide on different pretreatments for stent implantation. For example, for some calcified lesions with hard plaque properties, operators can use cutting balloon, scoring balloon or double guide wire balloon for pre-stent implantation, if necessary, using rotational atherectomy or excimer laser coronary atherectomy or intravscular lithotripsy. Kim et al. based on optimization criteria [minimum stent area (MSA) ≥ 5.5 mm^2^ or 80% of mean reference lumen area (MLA)], conducted four randomized trials comparing IVUS and angiographic guidance in long *de novo* lesions (≥ 26 mm) or chronic total occlusion lesions. A total of 1,396 patients who received an IVUS-guided intervention were enrolled and divided into two groups (stent-optimized and non-optimized). A significant number of patients receiving IVUS did not meet the criteria for stent optimization. Age ≥ 72 years, lesion length ≥ 39 mm, and stent diameter < 3.0 mm were independent risk factors for stent non-optimization. Using IVUS found that the proximal and distal vascular lumen area of the reference segment was smaller in the non-optimized group. MSA in the non-optimized group was significantly lower than that in the optimized group. So as for MACEs, IVUS guidance is superior to CAG guidance in DES implantation. The MACE rate in the IVUS-optimized stent implantation group was significantly lower than that in the non-optimized group (12). Hong et al. integrated IVUS-XPL and ULTIMATE databases on IVUS versus CAG-guided PCI of long *de novo* lesions, and conducted 3-year follow-up to evaluate the differences in MACEs, ST, and TLR between the two groups. In addition, the differences between the IVUS-guided stent-optimized group and the non-optimized group were analyzed. This study demonstrated IVUS optimization guiding PCI was associated with reduced hard clinical endpoints and the need for repeated revascularization. Although the primary endpoint of cardiac death was lower in the IVUS-optimized PCI group than in the non-optimized group and the difference was not statistically significant. The results may be due to the small number of patients undergoing IVUS-guided DES implantation and the relatively small number of events (5).

In previous meta-analyses, IVUS-guided PCI reduced the incidence of MACEs in patients with complex lesions including bifurcation, left main artery disease and chronic obstructive disease (11–14). In recent meta-analysis, IVUS-guided PCI therapy for complex lesions played a better role in reducing the incidence of MACE, TLR, and TVR (15). The beneficial effects of IVUS are not limited to reducing the incidence of MACE. It can also reduce clinical events such as stent thrombosis and death in coronary drug-eluting stent (DES) implantation (16). However, in our study, it was found that IVUS-guided PCI could not reduce stent thrombosis and death in patients with long *de novo* coronary lesions. The results may be due to the small number of patients undergoing IVUS-guided DES implantation and the relatively small number of events. Similarly, in some complex high-risk percutaneous coronary intervention, for example, in patients with bifurcations or unprotected left main treated with ultrathin stents, short overlap final kissing balloon (FKI) guided by IVUS is associated with less restenosis. In a double-stent strategy, FKI was associated with less target vessel revascularization and restenosis (17).

### Limitations

The article has some acknowledged limitations. Less RCTs, small sample size and differences in trial duration may affect meta-analysis. In addition, in the ULTIMATE study, the long-term MACEs of IVUS-guided PCI and CAG-guided PCI in long lesions were not separately listed, so we conclude these two studies in our meta-analysis. The criteria for stent optimization are not completely uniform at present, which may affect the long-term differences between the IVUS guidance and CAG guidance.

## Conclusion

In our meta-analysis, we concluded that intravascular ultrasound (IVUS)-guided PCI improves long outcomes of patients than angiographic-guided PCI in patients with long *de novo* coronary lesions.

## Data availability statement

The original contributions presented in the study are included in the article/supplementary material, further inquiries can be directed to the corresponding author.

## Author contributions

SW and XW: concept and design. YW (third author): supervision and critical review. YW (fifth author) and XJL: analysis and interpretation. SW and CZL: drafting the manuscript and literature search. All authors contributed to fundings and approved the submitted version.
